# Predictors of Extracapsular Extension in Patients With Squamous Cell Carcinoma of the Head and Neck and Outcome Analysis

**DOI:** 10.7759/cureus.16680

**Published:** 2021-07-28

**Authors:** Toms Vengaloor Thomas, Madhava R Kanakamedala, Eldrin Bhanat, Anu Abraham, Eswar Mundra, Ashley A Albert, Shankar Giri, Rahul Bhandari, Srinivasan Vijayakumar

**Affiliations:** 1 Radiation Oncology, University of Mississippi Medical Center, Jackson, USA; 2 Radiation Oncology, Texas Oncology, Amarillo, USA; 3 Orthopaedic Surgery, University of Mississippi Medical Center, Jackson, USA; 4 Pathology, Universtiy of Mississippi Medical Center, Jackson, USA; 5 Radiation Oncology, G.V. (Sonny) Montgomery VA Medical Center, Jackson, USA

**Keywords:** extracapsular extension, necrosis, head and neck cancer, outcomes, predicting ece, lymph node necrosis

## Abstract

Introduction

Extracapsular extension (ECE) in the lymph nodes for patients with head and neck cancer has been found to be a poor prognostic factor in multiple studies. The purpose of the study is to evaluate the predictive factors for ECE on computer tomography (CT) imaging for patients undergoing surgery and to analyze outcomes.

Methods

We conducted an Institutional Review Board-approved, Health Insurance Portability and Accountability Act (HIPAA)-compliant retrospective review of 82 patients with biopsy-proven squamous cell carcinomas of the head and neck who underwent definitive surgery without neoadjuvant chemotherapy or radiation therapy. CT scans were evaluated for the level of involvement, size, and presence or absence of central necrosis. Extracapsular extension in lymph nodes on the postoperative pathology was correlated with the central necrosis in the lymph nodes appreciated on the CT neck with contrast. Survival estimates were evaluated using the Kaplan-Meier test.

Results

ECE on postoperative pathology was seen in 74.07% of patients who had evidence of central necrosis in lymph nodes on preoperative CT neck compared to 46.43% without CT necrosis (p=0.013). The incidence of ECE is higher in poorly differentiated tumors and also nodal stages >N2c at presentation. Patents with ECE had inferior disease-free and overall survival (OS).

Conclusions

Our results reveal that patients with necrosis on CT and with moderately to poorly differentiated tumors have a high incidence of extracapsular extension. There was no difference in local control (LC) between the groups of patients, but the OS was inferior in patients with ECE. Predicting extracapsular extension upfront helps to formulate the appropriate treatment. We propose to study additional chemotherapy to improve outcomes in patients with positive extracapsular extension.

## Introduction

Peters et al. studied the criteria predicting the risk of local recurrence in the neck after surgery in patients with squamous cell carcinoma of the head and neck [1]. In their multivariate analysis, extracapsular extension (ECE) in lymph nodes was the single most important prognostic variable predicting treatment failure in the neck. In this randomized trial, the local control improved in the high-risk group with the addition of adjuvant radiation therapy escalating the dose to 63 Gray (Gy). Subsequent trials categorized those patients with extracapsular extension into the high-risk category. 

Both the European organization for Research and Treatment of Cancer (EORTC) and the Radiation Therapy Oncology Group (RTOG) conducted randomized trials in which patients with high-risk features were randomized to either radiation therapy alone versus concurrent chemo-radiation therapy (CRT) [2, 3]. Both these trials confirmed improvements in loco-regional controls with the addition of chemotherapy to radiation therapy in high-risk patients, even though the criteria for inclusion were not similar in these trials. A subsequent meta-analysis that combined RTOG and EORTC studies revealed that margin positivity and extracapsular involvement were the two factors that benefitted from adding chemotherapy to radiation therapy [4]. 

As previously reported, ECE is highly correlated with the size of the lymph nodes, but nodes less than 1 cm may also harbor ECE at a rate as high as 25% [5, 6]. Several studies evaluated the utility of Computed Tomography (CT) scans in diagnosing the ECE, but these were with negatively reported results [7-13].

## Materials and methods

**Figure 1:**
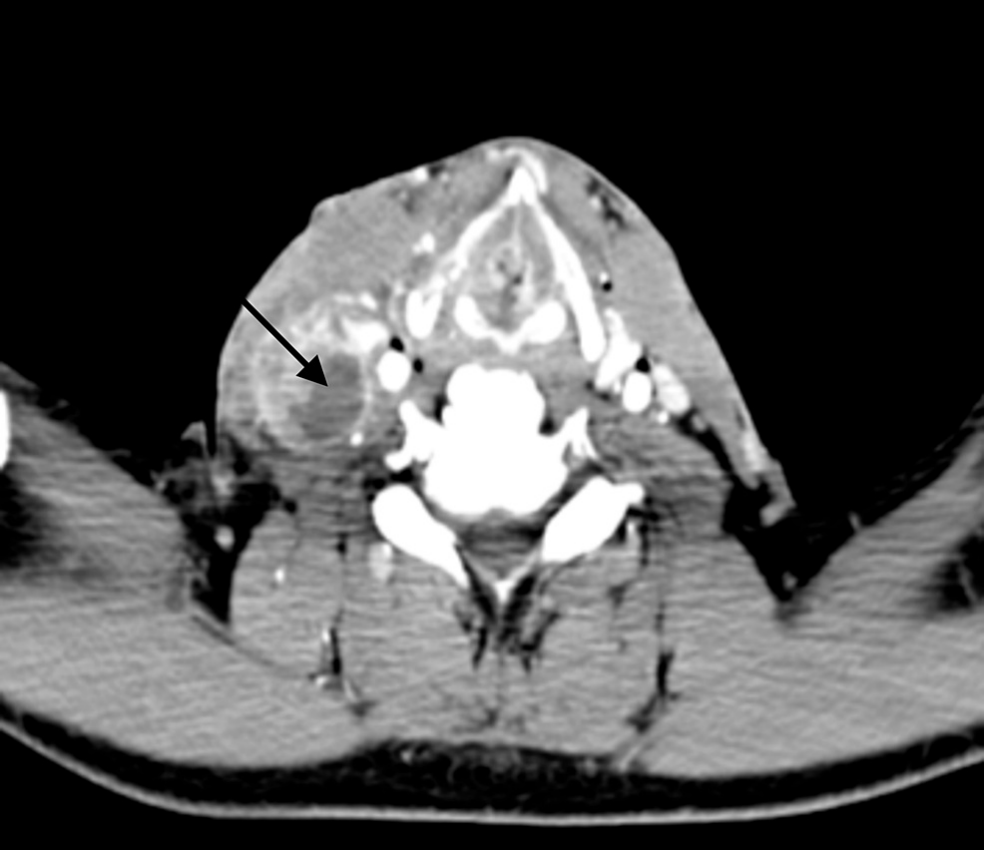
Computed tomography images of necrotic lymph nodes (necrotic lymph nodes in right level 3). Black arrow pointing towards the necrosis.

**Figure 2:**
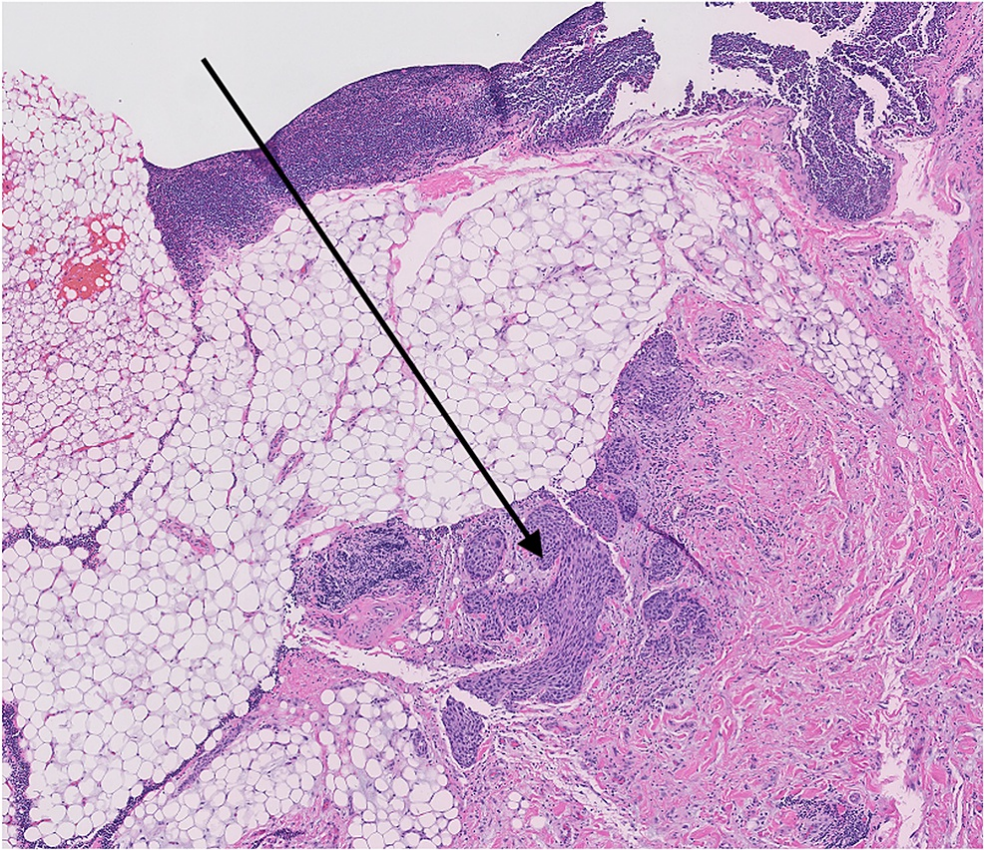
Extracapsular extension in lymph nodes on post-operative pathology (high power 10x).

**Figure 3:**
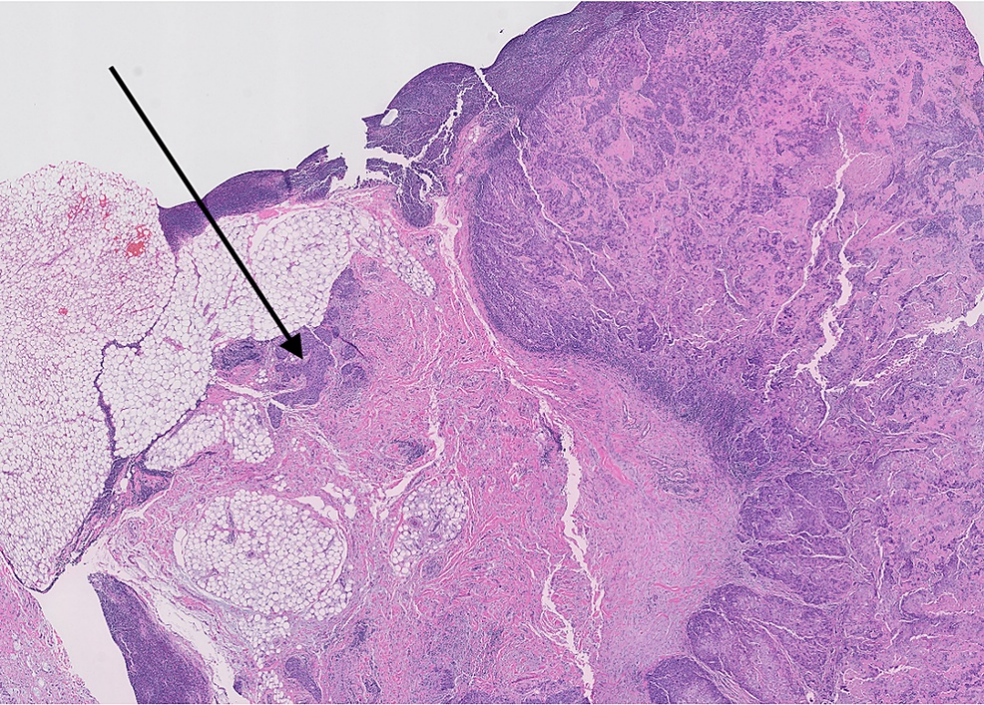
Extracapsular extension in lymph nodes on post-operative pathology (low power, 2x).

At the University of Mississippi Medical Center, we retrospectively evaluated 82 patients diagnosed with squamous cell carcinomas of the head and neck, treated between 2008 and 2014. Institutional review board approval was obtained for a retrospective review of these patients. Initial staging at diagnosis was performed utilizing CT neck with contrast. CT scans were also evaluated for the level of involvement, size, and presence or absence of central necrosis (Figure 1). All patients underwent surgical resection of primary cancer with neck dissection as a means of definitive treatment, without any neoadjuvant chemotherapy or radiation therapy. Extracapsular extension in lymph nodes on the postoperative pathology (Figures 2, 3) was correlated with the central necrosis in the lymph nodes appreciated on the CT neck with contrast. Patients were treated with either radiation therapy alone or concurrent CRT after the surgery, depending on the postoperative pathological risk factors.

Radiotherapy was delivered utilizing Intensity Modulated Radiation Therapy/Volumetric Modulated Arc Therapy (IMRT/VMAT) techniques. After completing the adjuvant treatment, the patients were followed up per National Comprehensive Cancer Network (NCCN) guidelines.

SPSS Statistics version 23 (IBM Corp., Armonk, USA) was used for statistical analysis. The cross-associations between variables were tested using the cross tabs function. A Chi-Square test was used between categorical variables to justify and estimate the significance of the association. The survival estimates for overall survival, disease-free survival, and distant metastasis-free survival were evaluated using the Kaplan-Meier test. Logrank test was used to estimate the level of significance in these tests. Cox regression using backward elimination was used to determine the significant covariates in the calculation of survival.

## Results

Of the 82 patients, 33 patients had an oral cavity primary, six with oropharynx, 30 with larynx, six with hypopharynx, six with skin, and one with an unknown primary. Based on the American Joint Committee on Cancer (AJCC) nodal staging 7th edition, 13 patients were grouped into N1, four patients into N2a, 25 patients into N2b, 35 patients into N2c, and five patients had N3 disease. The percentage of nodes with ECE based on the nodal stage was evaluated by substages (Table 1). ECE incidence increases with the N stage, with 20.73% positive ECE in N1+N2a, 60% in N2b, 80% in N2c, and 100% in N3 nodal disease. 

**Table 1:** Extracapsular extension (ECE) and its relation to nodal stage and nodal size.

Nodal Stage	Number of patients (82)	ECE: Number (%)
N1+N2a	17/82	7/17 (20.73%)
N2b	25/82	15/25 (60%)
N2c	35/82	28/35 (80%)
N3	5/82	5/5 (100%)
Nodal Size		
<3 cm	57	33/57 (57.90%)
>3cm	25	20/25 (80%)

Overall, the median size of the nodes was 2.5 cm (range 0.3-6.5 cm). As can be seen in Table 1, we analyzed the incidence of ECE based on nodal size. Prevalence of ECE in lymph nodes of size <3 cm was 57.9%, which proved to be worse with increasing nodal size. Specifically, there was an 80% prevalence of ECE in lymph nodes >3 cm.

Among 82 patients, 54 were found to have central necrosis in lymph nodes on CT imaging performed for staging. We correlated the extracapsular extension in the histopathology reports to the necrosis on the CT scans. ECE was positive in 46.4% of patients without necrosis on CT, whereas ECE was positive in 74.07% of patients with necrotic lymph nodes and was statistically significant (p=0.013). We found a similar correlation between necrosis and ECE, even in lymph nodes size <3 cm (n=57), as shown in Table 2. 

**Table 2:** Correlation of necrosis with extracapsular extension (ECE). ECE(+ve): extracapsular extension is present; ECE(-ve): extracapsular extension is absent; Necrosis(+ve): necrosis is present on the CT scan in the lymph node; Necrosis(-ve): necrosis is absent in the lymph node on CT scan.

All Nodes=82 pts		ECE(+ve)	ECE(-ve)	p-value
	Necrosis(-ve)=28	13/28 (46.43%)	15/28 (53.57%)	0.013
	Necrosis(+ve)=54	40/54 (74.07%)	14/54 (25.93%)	
Nodes <3 cm = 57 pts				
	Necrosis(-ve)=25	10/25 (40.00%)	15/25 (60.00%)	0.016
	Necrosis(+ve)=32	23/32 (71.88%)	9/32 (28.13%)	

Among the 82 patients under evaluation, only three patients had well-differentiated tumors, while 59 were moderately differentiated, and 20 were poorly differentiated, as seen in Table 3. The extracapsular extension incidence was high in tumors that were poorly differentiated at around 80%, resulting in no statistically significant difference in ECE positivity based on the presence or absence of necrosis in this histopathological group. However, among the moderately differentiated group, ECE was present in 38.4% of the lymph nodes without necrosis versus 74.29% in lymph nodes with necrosis. This finding shows a statistically significant increase in ECE in the moderately differentiated group with necrotic lymph nodes.

**Table 3:** Correlation of necrosis with extracapsular extension (ECE) as based on differentiation: moderately (G2) versus poorly (G3) differentiated. ECE(+ve): extracapsular extension is present; Necrosis(+ve): necrosis is present on the CT scan in the lymph node; Necrosis(-ve): necrosis is absent in the lymph node on CT scan.

Differentiation		ECE(+ve)	p-value
Moderate (N=59/82)	Necrosis(-ve)=24	10/24 (38.46%)	0.012
	Necrosis(+ve)=35	26/35 (74.29%)	
Poorly (N-=20/82)	Necrosis(-ve)=4	3/4 (75.00%)	0.531
	Necrosis(+ve)=16	14/16 (87.50%)	

Besides, we evaluated endpoints, including loco-regional failure (LRF), distant metastases (DM), and overall survival (OS) in patients who completed all the prescribed adjuvant treatment. We excluded 13 patients that were non-compliant with the adjuvant treatment and follow-up visits. Thus, a total of 69 patients were available in the final analysis. 

**Figure 4:**
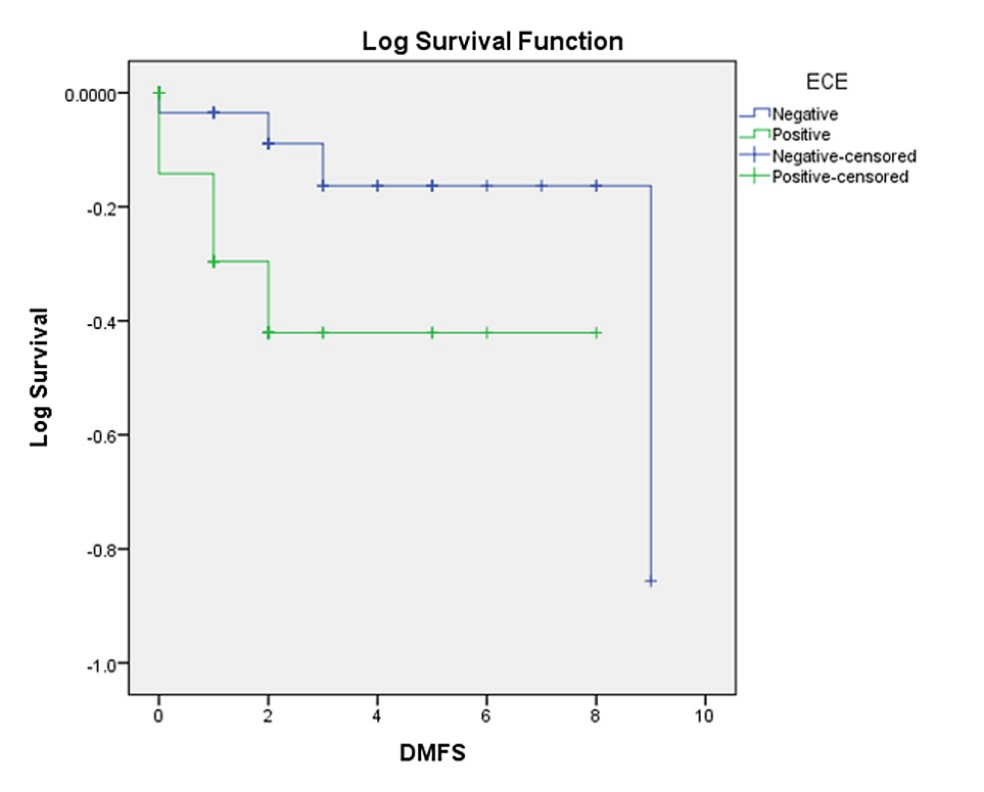
Kaplan-Meier distant metastasis-free survival (DMFS) curve for patients based on the presence (ECE+ve) or absence (ECE-ve) of extracapsular extension (ECE) of lymph nodes for head and neck cancer patients. ECE+ve: extracapsular extension present; ECE-ve: extracapsular extension absent.

**Figure 5:**
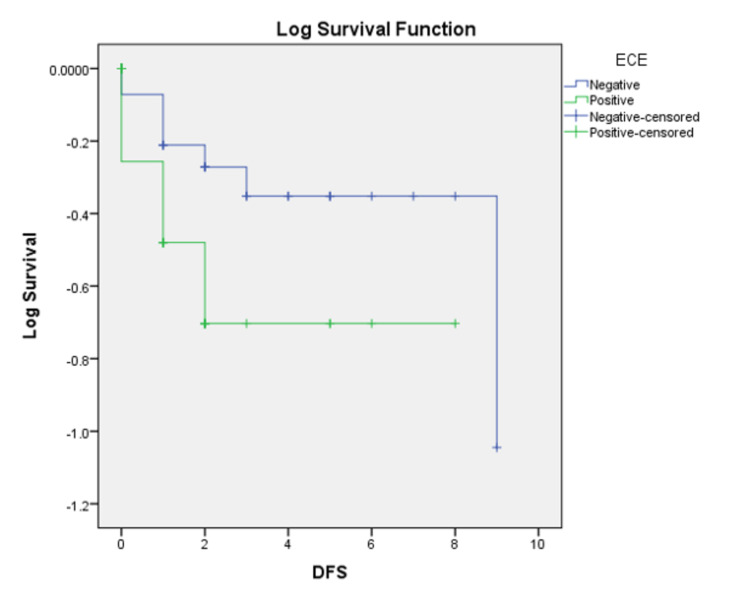
Kaplan- Meier disease-free survival (DFS) curve based on the presence (ECE+ve) or absence (ECE-ve) of extracapsular extension (EXE) of lymph nodes for head and neck cancer patients. ECE+ve: extracapsular extension present; ECE-ve: extracapsular extension absent.

**Figure 6:**
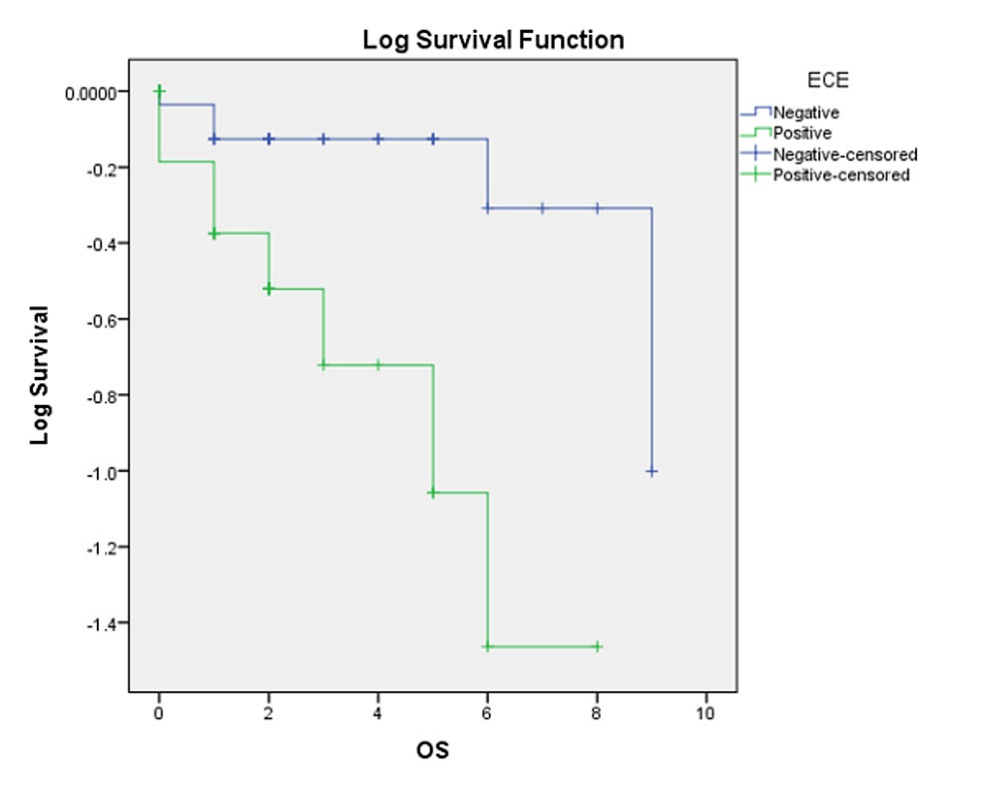
Kaplan-Meier overall survival (OS) curve based on the presence (ECE+ve) or absence (ECE-ve) of extracapsular extension (ECE) of lymph nodes for head and neck cancer patients. ECE+ve : extracapsular extension present; ECE-ve: extracapsular extension absent.

The median follow-up was five years. The loco-regional failures were similar in both groups (16 vs. 17.7%, p=0.811). However, the distant failures (Figure 4) were much higher in the ECE+ve group of 34.4%, whereas it was only 8.5% in the ECE-ve group (p=0.035). We observed a statistically significant decline in disease-free survival (DFS) (Figure 5) in the ECE+ve group compared to ECE-ve (49.5% vs. 76.2%, p=0.048), translating to decrement in the OS (59.4% in ECE+ve vs. 88.2% in ECE-ve, p=0.02) (Figure 6). Table 4 summarizes the outcomes.

**Table 4:** Outcome analysis based on extracapsular extension (ECE). ECE+ve: extracapsular extension present; ECE-ve: extracapsular extension absent.

Outcomes	ECE(-ve)	ECE(+ve)	P-value
Loco Regional Failure	16 %	17.7 %	0.811
Distant Failure	8.5 %	34.4 %	0.035
Disease-Free Survival	76.2 %	49.5 %	0.048
Overall Survival	88.2 %	59.4 %	0.02

## Discussion

Necrosis can be defined as cell death caused by loss of membrane integrity, intracellular organelle swelling, and adenosine triphosphate (ATP) depletion leading to an influx of calcium. Necrosis involves rapid and passive cell death. Necrosis is associated with the loss of plasma membrane integrity, swelling, and bursting of groups of cells rather than single cells at the cellular level. Necrosis is typically attributed to tumors with aggressive biology, as they have been found to outgrow their blood supply quickly [14]. Generally, there is an increased demand for nutrients and oxygen in the tumor microenvironment. Tumor hypoxia and depletion of energy in tumors create an acidic environment that leads to extrinsic activation of immune cells to release cytotoxins, which results in necrosis and paradoxically enhanced tumor growth [15]. The ability of nodal metastases to recruit degradation factors that permit the tumor to break through the lymph node capsule represents a very aggressive tumor biology [16, 17]. 

In our study, we found a statistically significant correlation between necrosis in lymph nodes on CT and the presence of extracapsular extension on postoperative pathology (74% vs. 46% for lymph nodes with and without necrosis, respectively). This finding was true even in smaller lymph nodes, size <3 cm. This retrospective review confirms the results from earlier studies that necrosis correlates with ECE in a larger patient population [18, 19].

When the data is further analyzed based on differentiation, a significant correlation was observed in patients with moderate differentiation (74% vs. 38% for lymph nodes with and without necrosis on lymph nodes, respectively), but not in poorly differentiated histology patients. However, ECE incidence was high in poorly differentiated (82.6%) compared to moderately differentiated histology (53.4%). This finding proves that poorly differentiated histology itself denotes aggressive tumor biology, thus increasing the risk of ECE with or without central necrosis on the preoperative imaging. 

More than 80% of patients with neck nodal stage >N2c, poorly differentiated histology, and nodal size >3 cm had ECE. Predicting extracapsular extension becomes valuable information for clinicians planning treatment [20-23]. Therefore, in patients who are at high risk for having an extracapsular extension of tumor in the lymph nodes, it is better to be treated with definitive CRT rather than the combined modality of surgery followed by adjuvant CRT. We acknowledge that the American Joint Committee on Cancer (AJCC) 8th edition staging system has incorporated ECE into the staging system [24].

## Conclusions

Our results reveal that patients with necrosis on CT and with moderately to poorly differentiated tumors have a high incidence of extracapsular extension. There was no difference in local control (LC) between the groups of patients, but the OS was inferior in patients with ECE, which can be explained by the higher incidence of distant metastases in this group. As the development of distant metastases was higher in patients with ECE, we propose to study administering additional chemotherapy in this group of patients to prevent distant metastases and improve overall survival. 
